# Detection of a novel gross deletion in the *UNC13D* gene ends the diagnostic odyssey for a family with familial hemophagocytic lymphohistiocytosis 3

**DOI:** 10.1186/s12887-023-04510-3

**Published:** 2024-01-11

**Authors:** Chinmayee B. Nagaraj, Diana S. Brightman, Hannah Rea, Emily Wakefield, Nina V. G. Harkavy, Lisa Dyer, Wenying Zhang

**Affiliations:** 1 Division of Human Genetics, Cincinnati Children’s Hospital Medical Center, 3333 Burnet Avenue, MLC 7016, Cincinnati, OH 45229 USA; 2 Department of Pediatrics, University of Cincinnati College of Medicine, Cincinnati, OH USA; 3 Department of OB/GYN, Columbia University Vagelos College of Physicians and Surgeons, New York City, NY USA

**Keywords:** Familial hemophagocytic lymphohistiocytosis type 3, UNC13D, Deletion, Diagnostic odyssey

## Abstract

**Background:**

Familial hemophagocytic lymphohistiocytosis (FHL) is an immunological disorder characterized by overactivation of macrophages and T lymphocytes. This autosomal recessive condition has been characterized into multiple types depending on the genetic etiology. FHL type 3 is associated with bi-allelic pathogenic variants in the *UNC13D* gene.

**Case presentation:**

We present a 12-year diagnostic odyssey for a family with FHL that signifies the advances of FHL genetic testing in a clinical genetic diagnostic laboratory setting. We describe the first case of a large *UNC13D* gross deletion in *trans* to a nonsense variant in a family with FHL3, which may have been mediated by Alu elements within introns 12 and 25 of the *UNC13D* gene.

**Conclusions:**

This case highlights the importance of re-evaluating past genetic testing for a patient and family as test technology evolves in order to end a diagnostic odyssey.

## Background

Familial hemophagocytic lymphohistiocytosis (FHL) is an immunological disorder characterized by overactivation of macrophages and T lymphocytes. Organs, such as the liver and spleen, are affected by this uncontrolled immune response and patients typically require a haematopoietic stem cell transplantation. This autosomal recessive condition has been characterized into multiple types, with FHL type 3 being associated with biallelic pathogenic variants in the *UNC13D* gene [1, 2]. Currently, the two most common genes associated with FHL, *PRF1* (FHL2 OMIM # 603,553) and *UNC13D* (FHL3 OMIM # 608,897), account for around 70% of FHL cases in some populations [3, 4].

To date, there have been over 200 variants reported in *UNC13D* in association with FHL3, with the vast majority (> 99%) being missense, nonsense, small indels, or splicing variants that result in loss of function and can be detected by sequence analysis [5]. Gross deletions and duplications, as well as chromosome rearrangement, are far less common in the literature, with the only known gross duplication described as recurrent by recent reports [6, 7]. Meeths et al. [8] in 2011 reported a chromosome rearrangement event, a 253 kb inversion in *UNC13D*, in association with FHL3. The gross deletions in *UNC13D* that have been reported to date range from 24 to 90 base pairs [9–11] and are small enough to be detected by Sanger sequencing.

Here, we present a 12-year diagnostic odyssey for a family with FHL that signifies the importance of re-evaluating past genetic testing for a patient and family with inconclusive or negative results and the advances of FHL genetic testing technology in a clinical genetic diagnostic laboratory setting. In this case we identified a multi-exon gross deletion involving *UNC13D* in *trans* to a nonsense variant. To the best of our knowledge, this is the first multi-exon gross deletion reported in the *UNC13D* gene.

## Case presentation

**Fig. 1:**
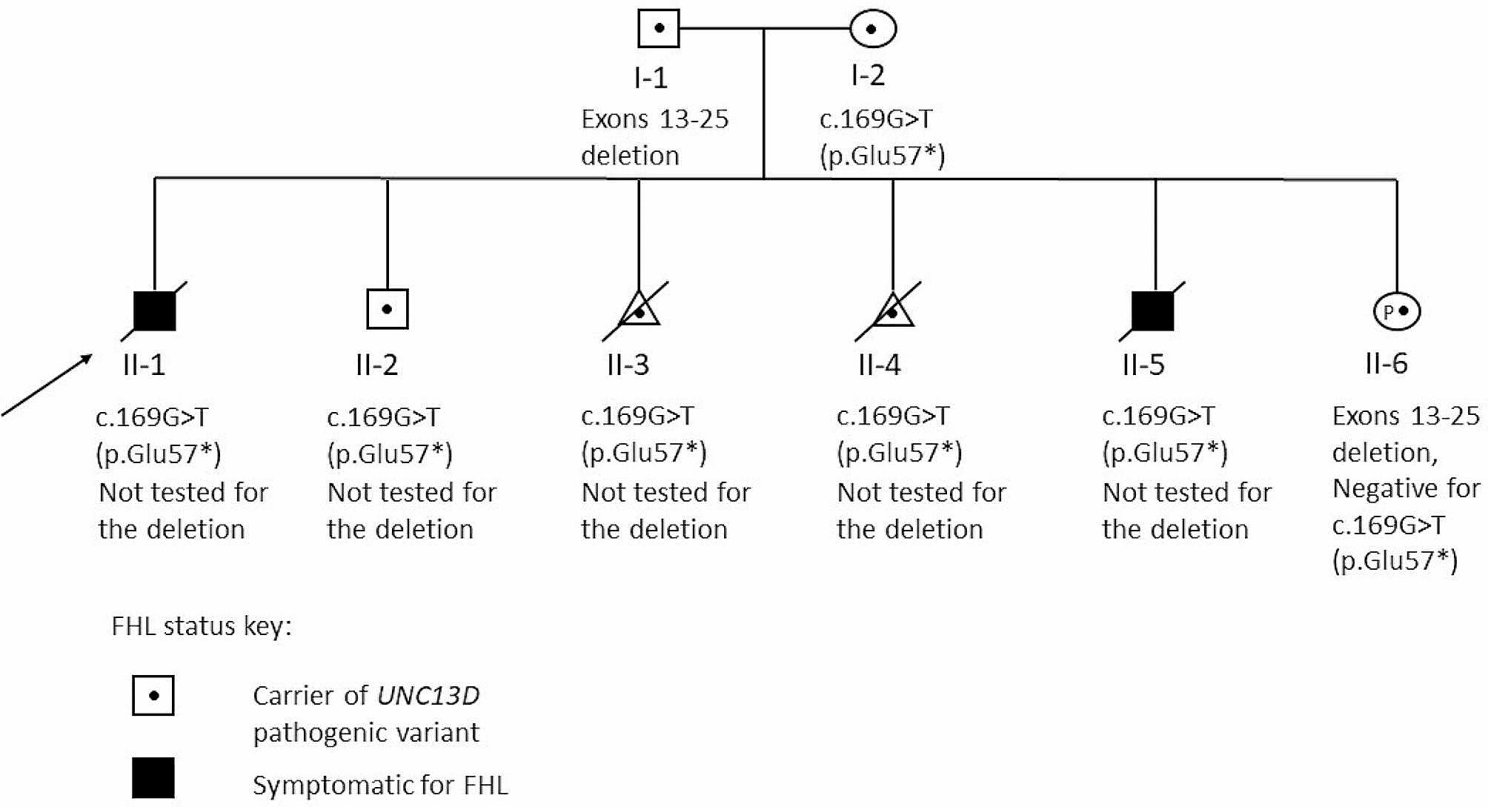
Pedigree of the family with *UNC13D* genetic test results

The proband (II-1) is the first born of a non-consanguineous couple (father, I-1; mother, I-2) (Fig. 1). The proband was born in 2006 and presented early in infancy with fevers, pancytopenia, and upregulated cytotoxic proteins in cytotoxic cells. The proband was clinically diagnosed with hemophagocytic lymphohistiocytosis (HLH) and passed away at the age of 4 months. Sanger sequencing of *PRF1, UNC13D*, and *SH2D1A* was performed posthumously using tissue sample at our clinical genetic diagnostic laboratory in 2006. The testing identified a heterozygous pathogenic nonsense variant in *UNC13D* and a heterozygous missense variant of uncertain significance (VUS) in *PRF1*, which still remains a VUS at this time. A second variant was not identified in either *UNC13D* or *PRF1* at that time. Parental *UNC13D* Sanger sequencing ordered at our laboratory by their maternal fetal medicine clinical team revealed both the *UNC13D* and *PRF1* variants to be maternally inherited.

The couple had a second child in 2008 (II-2) who has been healthy to date and had genetic testing in 2013 at our laboratory. This child was identified to be a carrier of the familial *UNC13D* nonsense variant and has been clinically monitored according to HLH protocol.

The couple had two pregnancies in 2010 (II-3 and II-4) and opted for prenatal exclusion testing for the familial *UNC13D* nonsense variant at our laboratory. Both pregnancies were positive for the *UNC13D* nonsense variant, putting them each at a 50% risk of FHL. The family chose to terminate the pregnancies.

The couple had another pregnancy in 2011 (II-5). Prenatal exclusion testing for the familial *UNC13D* variant performed at another laboratory, was positive for this pregnancy. The couple decided to proceed with this pregnancy and had the child, who was clinically diagnosed with HLH and passed away at the age of 4 years.

The couple was pregnant again in 2018 (II-6). At this time, the family and their providers considered additional testing options that were available since their previous testing to further understand the genetic etiology of the FHL diagnosis in the family. Additional testing on the father, which included sequencing of *PRF1, UNC13D, STX11, STXBP2, RAB27A* and exon-level deletion/duplication analysis by array comparative genomic hybridization (aCGH) of *UNC13D* at our laboratory, identified a novel partial *UNC13D* deletion involving exons 13–25. Exon-level aCGH deletion/duplication analysis of *UNC13D* was negative for the mother. Prenatal testing for the familial *UNC13D* nonsense variant performed at another laboratory was negative. The karyotype and microarray results of this pregnancy were negative for any clinically significant structural or copy number variations at another laboratory. Prenatal testing for the paternal *UNC13D* deletion by exon-level aCGH at our laboratory was positive. The result discrepancy between the exon-level aCGH of *UNC13D* and genomic microarray might be due to the difference in probe densities on the platforms and/or the number of consecutive missing probes considered to make an accurate deletion call. This *UNC13D* partial gene deletion, between 7.63 kb to 8.19 kb, was probably too small and beyond the limit of detection of the microarray test. Their asymptomatic child (II-2) has not been tested for the paternal *UNC13D* deletion to date.

## Genetic analysis

Sanger sequencing of *UNC13D* on the proband’s genomic DNA in 2006 included the entire coding regions and exon/intron boundaries. In 2018, Sanger sequencing of *UNC13D* on the father’s DNA additionally included the deep intronic c.118–308 region, as well as the allele specific analysis for the pathogenic 253 kb inversion, which were added to the assay in 2011 at our laboratory. The maternally inherited nonsense pathogenic variant identified in *UNC13D* is [Chr17(GRCh37): g.73839332G > T, NM_199242.2] c.169G > T p.(Glu57*) and missense VUS in *PRF1* is [Chr10(GRCh37): g.72358167G > A, NM_001083116.1] c.1310 C > T p.(Ala437Val).

**Fig. 2:**
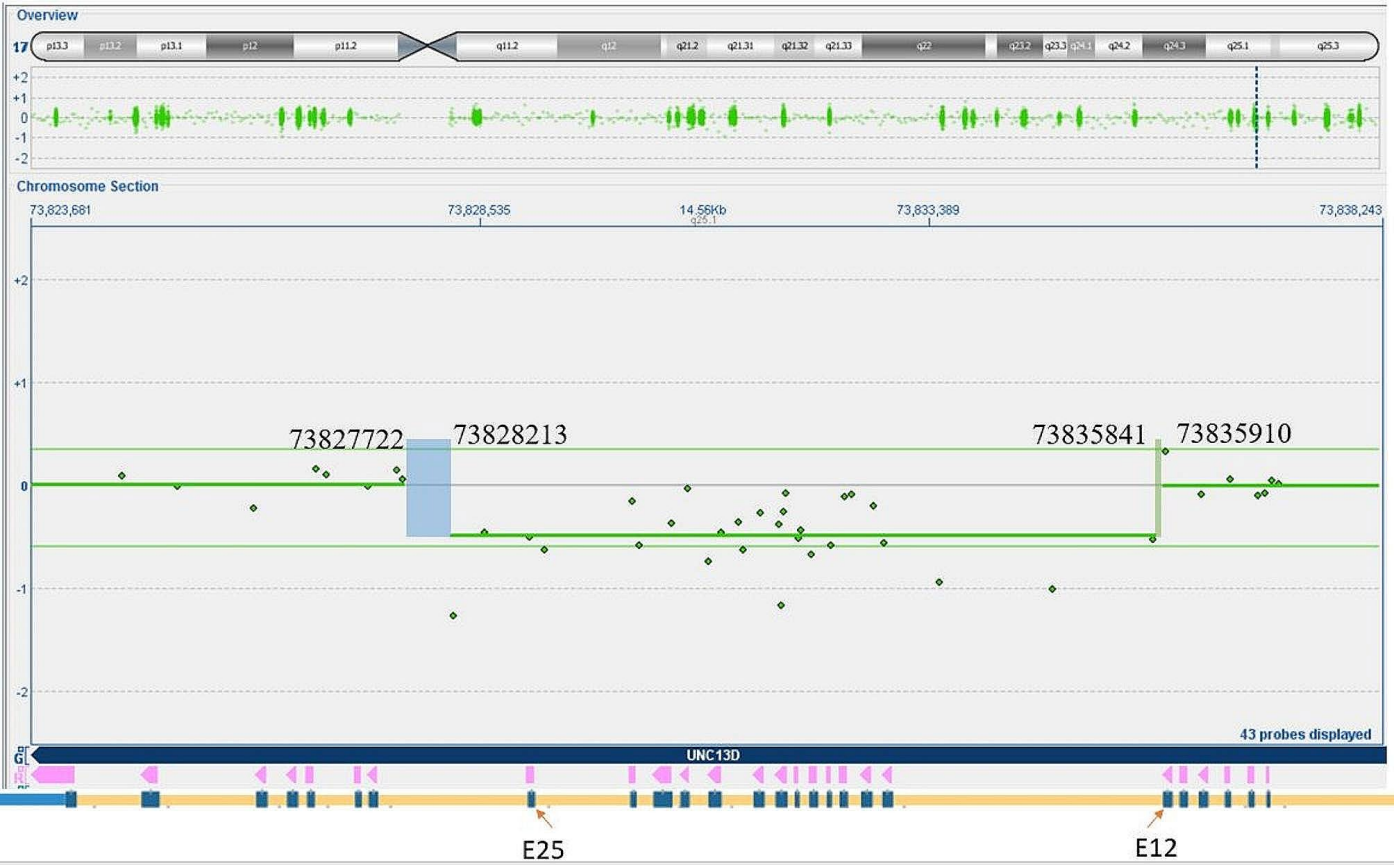
Deletion/ duplication by aCGH result with heterozygous partial gene deletion (intron 12 – intron 25) of *UNC13D*. The targeted deletion/duplication analysis of the *UNC13D* gene by aCGH on the father’s sample detected a heterozygous partial gene deletion in the *UNC13D* gene, which is between 7.63–8.19 kb and spans exon 13 to 25. As defined by the flanking probes, the breakpoint in intron 12 is expected to be between chromosome locations [hg19] chr17:73835841–73,835,910 (highlighted by light green rectangle) and the breakpoint in intron 25 is expected to be between chromosome locations [hg19] chr17:73827722–73,828,213 (highlighted by light blue rectangle). Please note, *UNC13D* is located on the minus strand of chromosome 17. The locations of E12 and E25 are highlighted by red arrows

The partial heterozygous deletion of the *UNC13D* gene that was identified in the father via exon-level aCGH involves exons 13–25 with breakpoints in introns 12 and 25 (Fig. 2). The deletion is between 7.63 kb and 8.19 kb in size and has the following minimum breakpoints, [hg19] chr17:73828213–73,835,841, and maximum breakpoints, [hg19] chr17:73827722–73,835,910. To the best of our knowledge, this is the first report of a multi-exon gross deletion of the *UNC13D* gene.

## Discussion and conclusions

This case highlights the importance of re-evaluation of genetic testing for patients with inconclusive or negative genetic testing results, especially in the context of evolving clinical genetic testing technologies. In 2006, our laboratory was the first and only clinical lab that offered genetic testing for HLH by Sanger sequencing in the USA. Although it was noted that large deletions or rearrangements in *UNC13D* would not be detected by this test method on the proband’s 2006 report, gene-specific deletion/duplication analysis of *UNC13D* was not available at that time in any laboratory in the USA. Detection of multiple consecutive homozygous single nucleotide polymorphisms (SNPs) and small deletions/duplications (small indels) within a region and/or evaluation of the variant zygosity concordance between the proband and parents would have suggested loss of heterozygosity due to a gross deletion (for example, if father appeared to be homozygous for a variant but the proband was wild type at that locus, this would have suggested that the father was actually hemizygous for that variant due to a gross deletion over the variant region on the other allele and the proband had inherited the gross deletion). However, no SNPs or small indels in *UNC13D* were detected in the proband to hint the presence of a gross deletion. Initial testing on the parents showed, other than the pathogenic nonsense variant detected in mother, there was only one heterozygous benign synonymous variant in exon 32, c.3198 A > G p.(Glu1066=), detected in father, which was not passed to the proband. Unfortunately, this information was not able to suggest the presence of any gross deletions and highlighted the test limitation of Sanger sequencing on detection of gross deletions/duplications. The testing for HLH has gradually become more comprehensive as the molecular genetic diagnostics have advanced. For instance, next-generation sequencing (NGS) as well as exon-level deletion/duplication through aCGH became clinically available around early 2010s and *UNC13D* deletion/duplication through aCGH was established in 2014 at our laboratory, 8 years after the proband had inconclusive testing in this case. The exon-level aCGH of *UNC13D* was ordered and performed in 2018 on father and eventually detected the gross deletion of exons 13–25 in this family.

Clinicians should be aware that deletion/duplication analysis may be crucial for diagnosis in some cases [12, 13], as well as the limitations for different testing methods. For example, NGS copy number variant (CNV) analysis, which has become more common, may miss some clinically significant CNVs, particularly deletions of less than three exons [14]. For HLH, recent studies have identified gross deletions and duplications in other genes related to HLH, including *PRF1*, *STX11*, and *STXBP2* [15–18]. In addition, large chromosome rearrangement events, such as the 253 kb *UNC13D* inversion, cannot be readily detected by Sanger sequencing, NGS, or aCGH, and require additional analysis method, such as PCR fragment analysis [8]. Therefore, it is important for clinicians to consider coverage of sequence variants, CNVs, and structural variants, through the assays of different labs and critically approach test coverage. We should also be careful about result interpretation; this family was previously reported as a possible example of digenic inheritance since a second pathogenic variant in *UNC13D* had not been identified [19].

We hypothesize that the deletion on the father’s allele may be mediated by Alu elements within introns 12 and 25 of the *UNC13D* gene. Alu elements contribute to almost 11% of human genome and are the most abundant mobile DNA elements [20]. Alu-mediated rearrangement is a significant contributor to evolution as well as human genetic disease. Hiejima et al. [6] described Alu elements within introns 6, 12, 25 and 30, and reported the first known in-frame intragenic duplication of *UNC13D* caused by aberrant recombination between the Alu elements within introns 6 and 12, which was reported as recurrent by Tomomasa et al. [7]. The pathogenic 253 kb inversion was also reported to be mediated by Alu elements located in intron 30 and the upstream region of *UNC13D* [8]. We suspect that the *UNC13D* deletion in this family may have been mediated by unequal crossing over of Alu elements or similar sequences within intron 12 and intron 25. This would be the second report of a CNV within the *UNC13D* gene mediated by Alu elements [6, 7]. These events, yet rare, reiterate the importance of thorough deletion/duplication testing in FHL3 cases when only one pathogenic allele has been identified.

Clinicians should periodically revisit genetic testing for patients with unsolved cases [21]. One limitation to continued evaluation is that the family may not present for care until another pregnancy is ongoing, which introduces time constraints. In addition, continuity of care may be disrupted when initial genetic testing was ordered for a proband who is now deceased. The family may next present for genetic testing when they see a maternal fetal medicine (MFM) specialist, OB/GYN, and/or prenatal genetic counselor who did not order the initial testing. We suggest that clinicians should periodically review all past genetic test results for patients with unsolved cases and should encourage these families to periodically contact them for updates. We also recommend that providers contact genetic testing labs to determine if any updates have been made to the variant classification for VUS and inquire if test updates or new technology is available. When possible, providers should send targeted testing to the same laboratory for all family members to ensure consistency and accuracy.

Herein we report the first multi-exon deletion of the *UNC13D* gene *in trans* to a nonsense sequence variant causing FHL type 3. This case highlights the importance of re-evaluating past genetic testing for a patient and family as test technology evolves in order to end a diagnostic odyssey.

## Data Availability

The datasets generated and/or analysed during the current study are available in the ClinVar repository, ID SCV003806696, https://www.ncbi.nlm.nih.gov/clinvar/variation/2442244/?oq=SCV003806696&m=Single+allele.
